# Electrocardiographic Abnormalities in Patients With Spinal Cord Injury With Deranged Lipid Profile

**DOI:** 10.7759/cureus.18246

**Published:** 2021-09-24

**Authors:** Muhammad Usman Shah Syed, Zunaira Khan, Arif Zulfiqar, Maleeha Ali Basham, Hafiz Abdul Haseeb, Saad Azizullah, Hebatalla Ismail, Mohammad Elbahnasawy, Zubia Nadeem, Sundas Karimi

**Affiliations:** 1 Internal Medicine, Dow University of Health Sciences, Karachi, PAK; 2 Accident and Emergency, Kingston Hospital, London, GBR; 3 Internal Medicine, Dow Medical College, Karachi, PAK; 4 Internal Medicine, Mayo Hospital, Lahore, PAK; 5 Medicine and Surgery, Royal College of Surgeons in Ireland, Dublin, IRL; 6 Internal Medicine, Alexandria Faculty of Medicine, Alexandria University, Alexandria, EGY; 7 Orthopedic Surgery, Dow University of Health Sciences, Karachi, PAK

**Keywords:** lipid profile, deranged lipid profile, cardiac abnormalities, ecg abnormalities, spinal cord injury

## Abstract

Introduction

Spinal cord injury (SCI) can lead to severe disability and neurogenic shock, arrhythmias, autonomic dysfunction, pressure ulcers, etc., of the autonomic nervous system. Therefore, in these patients, cardiovascular problems should be investigated frequently. This study was conducted to evaluate the electrocardiographic (ECG) abnormalities in patients with spinal cord injury having inappropriate lipid profiles and their relationship with each other.

Materials and methods

This cross-sectional study was held in the Internal Medicine Department of Mayo Hospital, Lahore, for a one-year duration from May 2020 to May 2021. It included 58 patients with spinal cord injury, 35 of whom had paraplegia, and 23 had tetraplegia. Fasting blood samples were taken for lipid profile analysis. Twelve-lead ECGs three times a day for one month were taken and analyzed in the context of previously available ECGs.

Results

Out of 58, the lipid profiles were found abnormal in 47 patients, 18 of whom had a normal ECG. The lipid profile was normal in 12, of which only one patient had ECG abnormalities. Cholesterol levels were found normal in 39 patients and deranged in 19 patients; low-density lipoproteins in nine patients, triglycerides in 18 patients, and high-density lipoprotein values in one patient were abnormal.

Conclusions

Sinus bradycardia was the most common ECG abnormality found in SCI patients with deranged lipid profiles. Further studies are needed in the future to validate the findings of this study.

## Introduction

Spinal cord injury (SCI) is a catastrophic, unfortunate, and final event that impairs a person's motor, sensory, and autonomic functions throughout his life [1,2]. Spinal cord injury causes severe disability, and these patients experience secondary complications such as neurogenic shock, arrhythmias, autonomic dysfunction, pressure ulcers, bowel and bladder dysfunction, chronic pain, anxiety, and urinary tract infections [3]. These patients are at greater risk of cardiovascular complications due to impaired mobility and impairment of the autonomic nervous system [3]. Often the most attention is paid to acute complications in their management, underestimating the chronic cardiovascular complications which are the leading cause of death and disease in patients with spinal cord injuries [3]. The electrocardiogram (ECG) is a suitable, accurate, and highly effective tool for the early diagnosis or detection of these problems. Abnormal ECG results are common after spinal cord injury due to arrhythmia or spinal dysautonomia [4]. ECG abnormalities in patients with chronic SCI have not yet been well studied. Previous studies have shown that the difference in ECG abnormalities in patients with SCI is due to the severity of the injury, the patient's age, or the degree of sympathetic disability [5,6]. Cardiovascular complications are very high in patients with spinal cord injuries due to a relatively sedentary posttrauma lifestyle, with increased sensitivity and incidence, with a predominance of other cardiovascular risk factors such as obesity, dyslipidemia, and diabetes [7,8]. Therefore, patients with spinal cord injury should be screened frequently compared to healthy people for cardiovascular problems. This study was designed to investigate the potential benefits of screening for electrocardiographic abnormalities in people with chronic SCI with a high-risk factor for cardiovascular diseases (CVD), in particular with an abnormal lipid profile.

## Materials and methods

Study setting and design

This study was conducted for a one-year duration from May 2020 to May 2021 in the Internal Medicine Department of Mayo Hospital, Lahore. It was a cross-sectional study.

Sample size, exclusion, and inclusion criteria

This study included 58 patients with spinal cord injury, 35 of whom had paraplegia, and 23 had tetraplegia. Patients with prespinal cord injury heart disease were not included in this study. Before the study, informed consent was obtained from all the patients.

Data collection

All patients were sampled early in the morning on an empty stomach for at least 14 hours for lipid profile analysis. All essential requirements for lipid profile testing were fully met before taking blood samples. Total cholesterol, high-density lipoprotein, low-density lipoprotein, and triglyceride levels were determined in the lipid profile. The ECG abnormalities were not diagnosed from a single ECG reading, but ECGs three times in one day for one month were taken in the supine position for at least 10 minutes and compared with at least two previous ECGs. This was done to reject the unexpected discovery on the ECG and confirm the findings.

Statistical analysis

All statistical analysis was performed using Statistical Package for Social Sciences (SPSS) version 23.0 (Armonk, NY: IBM Corp). Data were presented as mean and standard deviation for qualitative, whereas frequencies and percentages were recorded for quantitative variables.

## Results

Out of 58 patients, the lipid profiles were found abnormal in 47 patients, 18 of whom had a normal ECG, and the lipid profile was normal in 12 patients, of which only one patient had ECG abnormalities. Cholesterol levels were found normal in 39 patients and deranged in 19 patients; low-density lipoproteins in nine patients, triglycerides in 18 patients, and high-density lipoprotein values in one patient were abnormal, as shown in Table 1.

**Table 1:** The lipid abnormalities among patients with spinal cord injury. CH: Cholesterol, LDL: low-density lipoproteins, HDL: high-density lipoproteins, TG: triglycerides.

Results	CH	LDL	HDL	TG
Deranged	19	9	1	18
Normal	39	49	57	40
Total	58	58	58	58

The number of deranged parameters in the lipid profiles of the SCI patients is shown in Table 2.

**Table 2:** The number of parameters deranged in the lipid profiles of the SCI patients. SCI: spinal cord injury.

Number of parameters deranged	Number of patients
None of the parameters was deranged	11
Only one profile deranged	9
Only two profiles deranged	14
Three profiles deranged	9
All profiles deranged	0

Among the paraplegic patients, seven patients had ECG abnormalities, three of whom had partial right bundle branch block (RBBB), two had partial RBBB with sinus tachycardia, and two had sinus bradycardia. The remaining 28 had no ECG abnormalities. Among the patients with tetraplegia, 17 patients had ECG abnormalities, six of whom had sinus bradycardia, three had partial RBBB, two had partial RBBB with sinus bradycardia, two had left axis deviation, two had left ventricular hypertrophy, and two had a ventricular hypertrophy Q-wave inversion (V1-V4). The remaining six had no ECG abnormalities, as shown in Table 3.

**Table 3:** Cardiac abnormalities on ECG found in the SCI patients. ECG: electrocardiogram, SCI: spinal cord injury, RBBB: right bundle branch block.

ECG abnormalities found in the SCI patients	No. of patients
Paraplegics	35
Partial RBBB	3
Partial RBBB with sinus tachycardia	2
Sinus bradycardia	2
No abnormality detected	28
Tetraplegics	23
Sinus bradycardia	6
Partial RBBB	3
Partial RBBB with sinus bradycardia	2
Q-wave inversion (V1-V4)	2
Left ventricular hypertrophy	2
Left axis deviation	2
No abnormality detected	6
Grand total	58

During the regression analysis, the p-value corresponding to the above t statistic is 6.947 and the standard error of the regression coefficient is 0.09. Therefore, it is unlikely that the two variables are not related to each other. It also means that the coefficient value is correct (the lower the standard error, the higher the accuracy).

## Discussion

As the study was linked to a population rehabilitation center offering all possible rehabilitation facilities and resources, these patients are well managed and managed by physical therapists to prevent immobilization and exercise as well as possible. Along with this, they follow a strict plan of exercise and diet. It also helps them use a curved table and wheelchair ambulance, accompanied by daily breast and calf physiotherapy. Following a strict diet, proper exercise, and a responsible diet may result in different results in these studies. Sinus bradycardia was the most common abnormality in this population with a very low incidence of T-wave abnormalities. This is consistent with a previously performed study, which showed a similar frequency of ECG abnormalities in people after SCI compared to people without SCI [9-12]. In this study, there was no definitive evidence of myocardial ischemia or any abnormalities associated with coronary artery disease. Another study showed that T-wave abnormalities and overall morbidity were highest in SCI patients aged 40 to 49 years [13,14]. This study suggests that the risk of adverse outcomes also doubles in these patients. All ECGs in this study were taken in an ascending position to avoid the effect that body position could have on the ECG. Some ECG abnormalities, such as sinus stretching, can be attributed to the level of spinal cord injury and associated autonomic disability, but the remaining abnormalities cannot be fully explained by spinal cord injury and autonomic disability, as none of the participants in the study had an ECG injury or a medical condition [15,16]. Standard measures were used in all patients when the ECG was performed, and a number of ECG series were considered as confirmation of abnormalities. Despite the abnormalities associated with autonomic disease and residual lesion levels, there is not much of a difference in the incidence of other ECG abnormalities in these patients compared to the normal patient population. Due to spinal cord injury, these patients experience immobility, dysautonomia, and metabolism as additional risk factors [17,18]. Interestingly, patients with chronic SCI, especially those with longer trauma, have a higher risk of developing CVD compared to others. Therefore, these individuals should be closely monitored for the presence of chronic ischemia, and physicians who care for individuals with chronic SCI should include ECGs in their routine screening procedures. The SCI survivors are at increased risk of developing secondary debilitating conditions like deep vein thrombosis, urinary tract infections, muscle spasms, osteoporosis, pressure ulcers, chronic pain, and respiratory complications. While age is an important determinant of the risk of these disabilities, for people with chronic SCI, the duration of the injury is at least as important [19]. ECG abnormalities in patients with SCI were not very significant for ischemic lesions [20]. Some abnormalities result from the level of trauma and autonomic dysfunction. The ECG abnormalities do not differ much from the normal population, suggesting that the ECG is of similar value as a screening tool in both populations [21].

## Conclusions

Sinus bradycardia was the most common abnormality found on ECG in patients with SCI and deranged lipid profile. T-wave abnormality suggesting possible myocardial ischemia was found in only one patient. Further studies on a large scale are needed in the future to potentiate the results of this study and to see the benefits of screening for ECG abnormalities in these patients.

## References

[1] Ballestri S, Mantovani A, Nascimbeni F, Lugari S, Lonardo A (2019). Extra-hepatic manifestations and complications of nonalcoholic fatty liver disease. Future Med Chem.

[2] Akinlade OM, Owoyele B, Soladoye OA (2021). Carvedilol improves heart rate variability indices, biomarkers but not cardiac nerve density in streptozotocin-induced T2DM model of diabetic cardiac autonomic neuropathy [PREPRINT]. J Basic Clin Physiol Pharmacol.

[3] Lucci VM, Inskip JA, McGrath MS, Ruiz I, Lee R, Kwon BK, Claydon VE (2021). Longitudinal assessment of autonomic function during the acute phase of spinal cord injury: use of low-frequency blood pressure variability as a quantitative measure of autonomic function. J Neurotrauma.

[4] Lotter JK, Henderson CE, Plawecki A (2020). Task-specific versus impairment-based training on locomotor performance in individuals with chronic spinal cord injury: a randomized crossover study. Neurorehabil Neural Repair.

[5] Wecht JM, Harel NY, Guest J (2020). Cardiovascular autonomic dysfunction in spinal cord injury: epidemiology, diagnosis, and management. Semin Neurol.

[6] Krhut J, Wohlfahrt P, Pudich J (2021). Cardiovascular safety of mirabegron in individuals treated for spinal cord injury- or multiple sclerosis-induced neurogenic detrusor overactivity. Int Urol Nephrol.

[7] Fehlings MG, Chen Y, Aarabi B (2021). A randomized controlled trial of local delivery of a Rho inhibitor (VX-210) in patients with acute traumatic cervical spinal cord injury. J Neurotrauma.

[8] Mullen MT, McGarvey M (2021). Spinal cord infarction: clinical presentation and diagnosis. UpToDate.

[9] Trbovich M, Romo T, Polk M (2021). The treatment of neurogenic lower urinary tract dysfunction in persons with spinal cord injury: An open label, pilot study of anticholinergic agent vs. mirabegron to evaluate cognitive impact and efficacy. Spinal Cord Ser Cases.

[10] Morgan S (2020). Recognition and management of autonomic dysreflexia in patients with a spinal cord injury. Emerg Nurse.

[11] Wang S, Wecht JM, Legg Ditterline B (2020). Heart rate and blood pressure response improve the prediction of orthostatic cardiovascular dysregulation in persons with chronic spinal cord injury. Physiol Rep.

[12] Dorey TW, Walter M, Krassioukov AV (2021). Reduced reflex autonomic responses following intradetrusor onabotulinumtoxinA injections: a pre/post study in individuals with cervical and upper thoracic spinal cord injury. medRxiv.

[13] Stampas A, Zhu L, Li S (2019). Heart rate variability in spinal cord injury: asymptomatic orthostatic hypotension is a confounding variable. Neurosci Lett.

[14] Kim HS, Park JH, Lee HS (2021). Effects of wearable powered exoskeletal training on functional mobility, physiological health and quality of life in non-ambulatory spinal cord injury patients. J Korean Med Sci.

[15] Lin AP (2019). Overcoming technical challenges of MR spectroscopy in chronic spinal cord injury. Radiology.

[16] Pizzolato C, Gunduz MA, Palipana D (2021). Non-invasive approaches to functional recovery after spinal cord injury: therapeutic targets and multimodal device interventions. Exp Neurol.

[17] Karri J, Li S, Chen YT, Stampas A, Li S (2021). Observations of autonomic variability following central neuromodulation for chronic neuropathic pain in spinal cord injury. Neuromodulation.

[18] Wang HC, Lin YT, Huang CC, Lin MC, Liaw MY, Lu CH (2021). Effects of respiratory muscle training on baroreflex sensitivity, respiratory function, and serum oxidative stress in acute cervical spinal cord injury. J Pers Med.

[19] Bloom O, Wecht JM, Legg Ditterline BE (2020). Prolonged targeted cardiovascular epidural stimulation improves immunological molecular profile: a case report in chronic severe spinal cord injury. Front Syst Neurosci.

[20] Batista LH, Domingues WJ, e Silva AD, Lopes KA, de Castro Amorim ML, Rossato M (2021). Heart rate variability responses determined by photoplethysmography in people with spinal cord injury. Biomed Signal Process Control.

[21] Panza GS, Guccione AA (2020). Effect of repeated locomotor training on ventilatory measures, perceived exertion and walking endurance in persons with motor incomplete spinal cord injury. Spinal Cord Ser Cases.

